# Glycerol Monolaurate Contributes to the Antimicrobial and Anti-inflammatory Activity of Human Milk

**DOI:** 10.1038/s41598-019-51130-y

**Published:** 2019-10-10

**Authors:** Patrick M. Schlievert, Samuel H. Kilgore, Keun Seok Seo, Donald Y. M. Leung

**Affiliations:** 1 Department of Microbiology and Immunology, Carver College of Medicine, University of Iowa, Iowa City, Iowa, 52242 USA; 2 Department of Basic Sciences, College of Veterinary Medicine, Mississippi State University, Mississippi State, Mississippi, 39762 USA; 3 Department of Pediatrics, National Jewish Health, Denver, Colorado 80206 USA

**Keywords:** Paediatric research, Infection, Acute inflammation

## Abstract

Human milk has antimicrobial compounds and immunomodulatory activities. We investigated glycerol monolaurate (GML) in human milk versus bovine milk and infant formula for antimicrobial and anti-inflammatory activities. Human milk contained approximately 3000 µg/ml of GML, compared to 150 μg/ml in bovine milk and none in infant formula. For bacteria tested (*Staphylococcus aureus*, *Bacillus subtilis*, *Clostridium perfringens*, *Escherichia coli*), except *Enterococcus faecalis*, human milk was more antimicrobial than bovine milk and formula. The *Enterococcus faecalis* strain, which was not inhibited, produced reutericyclin, which is an analogue of GML and functions as a growth stimulant in bacteria that produce it. Removal of GML and other lipophilic molecules from human milk by ethanol extraction resulted in a loss of antibacterial activity, which was restored by re-addition of GML. GML addition caused bovine milk to become antimicrobial. Human milk but not bovine milk or formula inhibited superantigen and bacterial-induced IL-8 production by model human epithelial cells. GML may contribute beneficially to human milk compared to bovine milk or infant formula.

## Introduction

Human milk contains many known antimicrobial and immunomodulatory molecules, including immunoglobulins, antimicrobial peptides, and fatty acids^1–5^. The cumulative effect of the properties of human milk, including ability of bacterial species to utilize human milk oligosaccharides, is selection for bacteria such as bifidobacteria, lactobacilli, and enterococci, genera of bacteria that have acids and hydrogen peroxide as major end products of metabolism^6–10^. The reduced pH caused by these bacteria, and additionally, by known and unknown components of human breast milk make it more difficult for potentially pathogenic organisms to colonize^6,7^; for example potentially pathogenic *Escherichia coli* are inhibited^6,7^. Human milk, including human milk oligosaccharides^11,12^, also may reduce the occurrence of allergic disorders, including asthma, food allergy, and atopic dermatitis, the latter two of which have been linked to *Staphylococcus aureus* colonization in young children^13–15^.

We and others have characterized glycerol monolaurate (GML) as a fatty acid monoester with broad antimicrobial and anti-inflammatory properties^16–21^. Lauric acid, one of the esterase cleavage products of GML, also has potent antimicrobial and anti-inflammatory properties, but these properties require nearly 400-fold more lauric acid than GML^21^. Other fatty acid monoesters have significantly reduced antimicrobial activity compared to GML^21^. The antimicrobial properties of GML and free fatty acids extend to nearly all Gram-positive bacterial species except certain lactobacilli, bifidobacteria, and enterococci^21,22^. These resistant bacteria appear to be positively selected for due to GML mimicking a quorum-sensing growth stimulant known as reutericyclin or related tetramic acids^21,23^. The ultimate bacterial killing effect by GML appears to be due to interference with plasma membrane functions, resulting in loss of potential difference across the membrane^23^. Lactobacilli that produce reutericyclin have been incorporated into probiotic capsules in attempt to increase lactobacilli in the gastrointestinal tract^24^.

GML also prevents harmful pro-inflammatory processes *in vivo* at mucosal surfaces^16–20,25^, although *in vitro* studies with purified GML show toxicity to tissue culture cells *in vitro* at concentrations ≥100 μg/ml^26–28^. The differences between *in vivo* versus *in vitro* activities has not been investigated, but may in part be related to the modulation of GML toxicity by human serum albumin^29^. Our studies have shown, for example, that inflammation at the human and non-human primate vaginal mucosa facilitates simian immunodeficiency virus infection in rhesus macaque monkeys and production of menstrual toxic shock syndrome^16,18–20,30^. The production of local inflammation initially depends on microbial stimulation of epithelial cells, among other cells, to produce pro-inflammatory chemokines, such as IL-8 and MIP-3α, which attract innate and adaptive immune cells into the submucosal areas and facilitating barrier disruption^19,20,30^. Our studies also show that vaginal pathogens, but not lactobacilli and latex beads, activate epithelial cells to produce many expected pro-inflammatory chemokines^16,19,22,30^. GML interferes with normal signal transduction in epithelial cells and locally-recruited immune cells through membrane effects, but does not kill the host cells *in vivo*^26–28^. It is noteworthy that a small percentage of women (1%) have reutericyclin-producing lactobacilli and enterococci vaginally, and in such cases, there are minimal to no other vaginal microbes present; one of these enterococci is used in our current studies^21^. This raises the possibility that reutericyclin and its possible analogue GML have similar effects, both in killing other bacteria and preventing harmful inflammation, in the gastrointestinal tract^21^. Reutericyclin is expensive to purchase and evaluate, whereas GML is inexpensive and readily available for use in tests.

The goals of this study were to determine: (1) the amount of GML present in whole human versus whole bovine milk and commercial formula, and (2) the possible contribution of GML to the antimicrobial and anti-inflammatory properties of human milk compared to bovine milk and commercial formula. Our hypothesis was that GML may be present in human milk samples but not bovine milk and commercial formula. Furthermore, GML presence, along with other factors, may inhibit the growth of pathogens and at the same time exhibit anti-inflammatory activity. Overall, these studies provide important data on the advantages of use of human milk for infants.

## Results

### GML quantified in human and bovine milk samples and commercial formula

**Figure 1:**
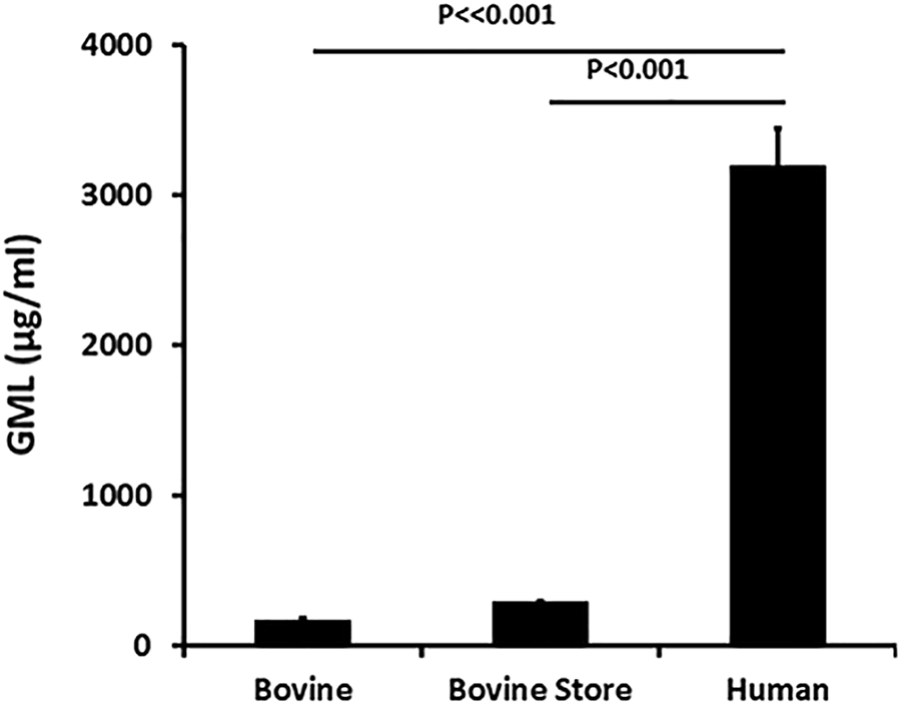
Glycerol monolaurate (GML) concentrations in 6 individual pasteurized whole bovine, 2 whole bovine pasteurized samples purchased from a local grocery store, and 4 pasteurized whole human milk samples. Data are recorded as mean ± SD. Student’s *t* test was used to assess differences in means. P ≪ 0.001 indicates mean differences much greater than p < 0.001.

Four human milk, 6 bovine whole milk from cows, and 2 store-purchased whole bovine milk samples were quantified for GML (Fig. 1). All milk samples had been pasteurized. Whether compared to whole bovine milk obtained from individual cows or to whole bovine milk purchased from a local grocery store, the four tested human milk samples had approximately 20 times more GML than the 8 bovine samples. There was minimal variation in GML concentrations in the human milk samples. Commercial infant formula (Similac^®^ Advance) contained no detectable GML.

### Effect of whole human versus bovine milk and formula on growth of bacteria

**Figure 2:**
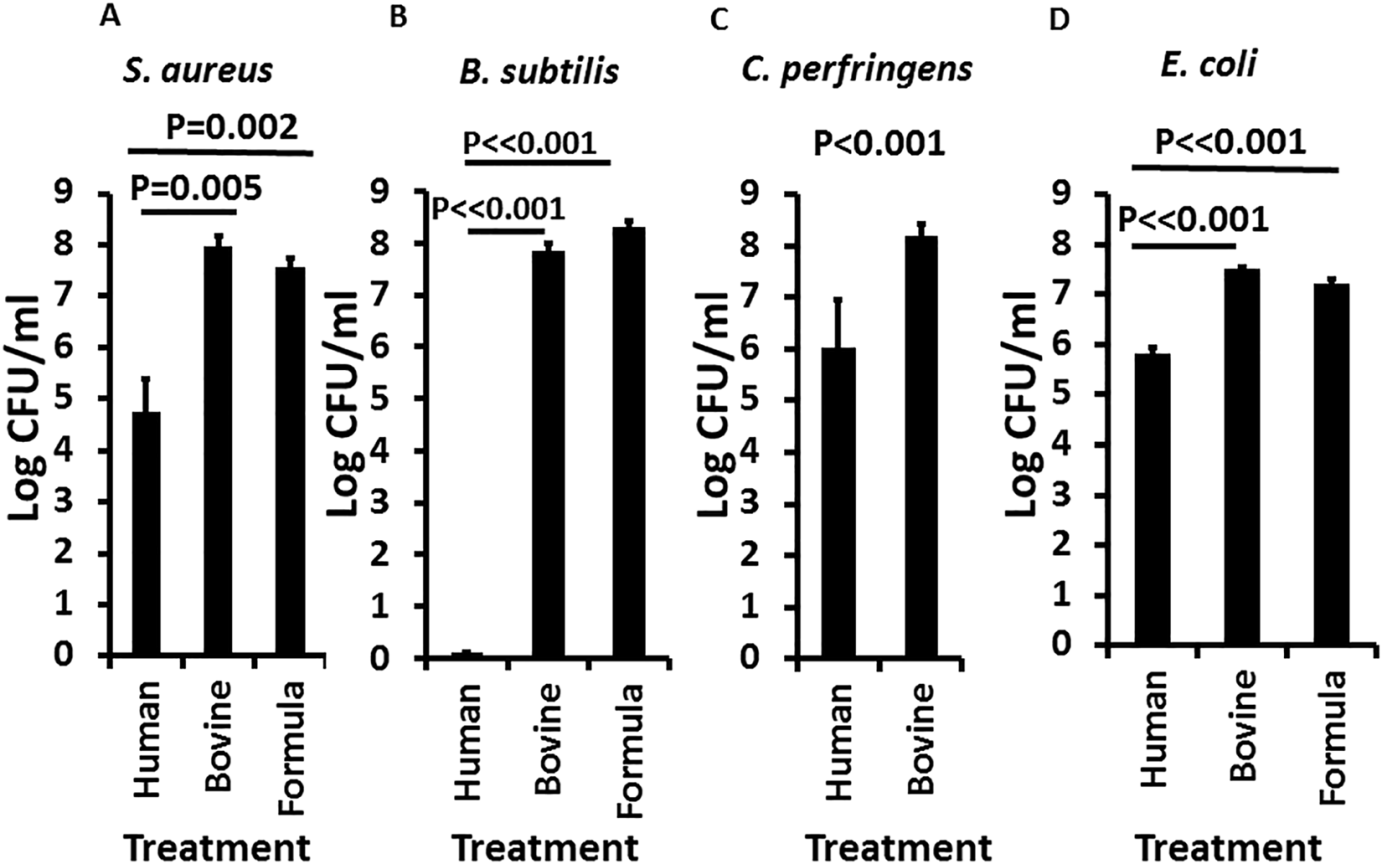
Effect of whole pasteurized human milk and bovine milk samples, and commercial infant formula on growth of (**A**) *S. aureus*, (**B**) *B. subtilis*, (**C**) *C. perfringens*, and (**D**) *E. coli*. Log colony-forming units (CFUs)/ml are shown for each treatment. Starting inoculum was 1–2 × 10^5^/ml. Samples were cultured to stationary phase for 24 hours with aeration (200 RPM; **A**,**B**,**D**) and stationary in an anaerobic chamber (**C**). Data represent means ± SD. Student’s *t* test was used to determine significant differences in means. P ≪ 0.001 indicates mean differences much greater than p < 0.001.

We tested the human milk, bovine milk samples, and the commercial infant formula sample for ability to inhibit the growth of selected Gram-positive aerobes *S. aureus* (a potential gastrointestinal pathogen known for its roles in food poisoning^31^ and enterocolitis^32,33^; Fig. 2A) and *B. subtilis* (highly susceptible to killing by GML^34^ and essentially a positive control; Fig. 2B); we tested human and bovine milk for effect on the Gram-positive anaerobe *C. perfringens* (anaerobe; potential gastrointestinal pathogen and microflora; Fig. 2C); and we tested human and bovine milk and commercial infant formula for effect on the Gram-negative *Escherichia coli* (potential gastrointestinal and urinary tract pathogen, microflora, representative of *Enterobacteriaceae*; Fig. 2D). For all three Gram-positive bacteria, the 4 human milk samples comparably inhibited the growth of the strains compared to the bovine milk samples and commercial formula. *B. subtilis* was more susceptible to killing by the human milk samples than either of the other two organisms; we previously observed the same differential susceptibility when we studied the effects of purified GML on Gram-positive bacteria^21,34^. Both aerobes and obligate anaerobes were significantly inhibited by human milk samples. There were only minimal differences in inhibitory activity among the human milk samples. The Gram-negative organism, *Escherichia coli*, which is clearly both a commensal and one of the leading causes of gastrointestinal and urinary tract infections worldwide, was likewise inhibited by human milk but not whole bovine milk or infant formula. It is likely that the effect on *E. coli*, and likely on other organisms depended on synergy among GML and other antimicrobial compounds in human milk; *E. coli* is typically not susceptible to GML alone, even at 3000 µg/ml^35^.

The stationary phases of all tested organisms, for comparison to milk and formula, in excellent growth medium (Todd Hewitt broth, Difco Laboratories, Detroit, MI) were: *S. aureus* MN8 7 × 10^9^/ml; *B. subtilis* 1 × 10^9^/ml; *C. perfringens* 5 × 10^8^/ml; and *E. coli* 2 × 10^9^/ml. Thus, the stationary phases of these organisms were generally 0.5–1 log higher in Todd Hewitt medium than whole bovine milk and infant formula.

**Figure 3:**
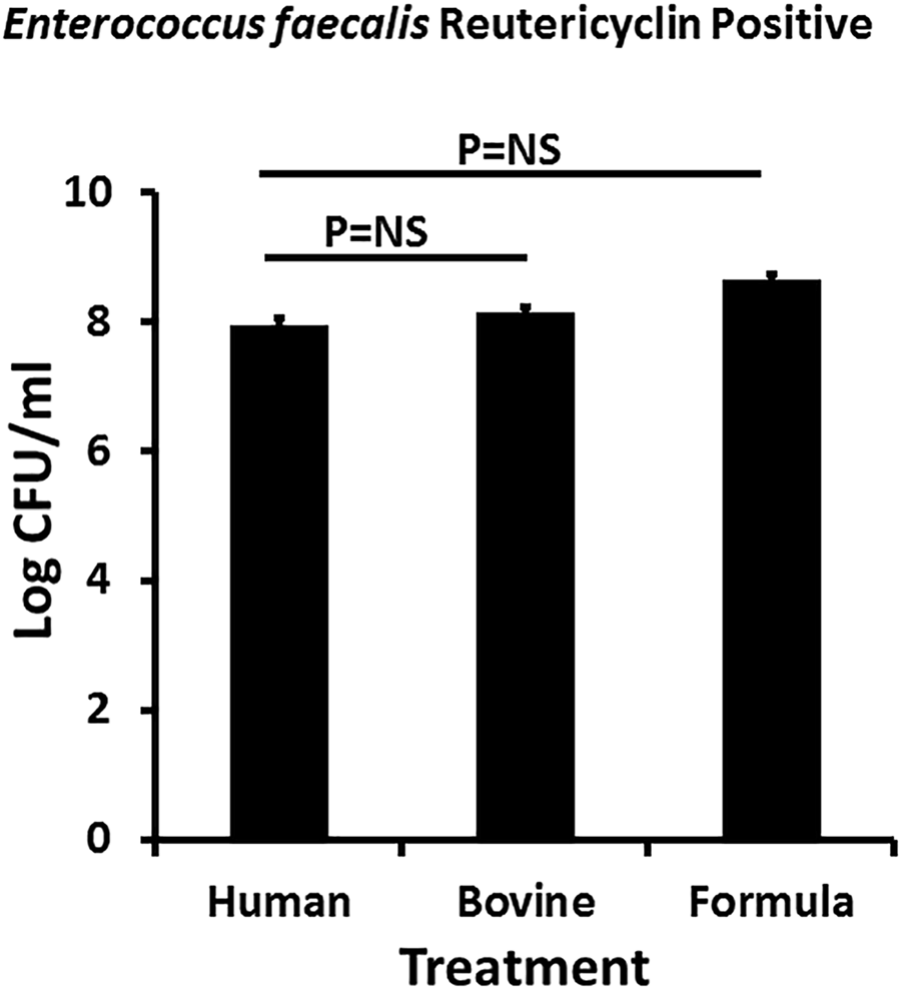
Effect of whole human and bovine milk and commercial infant formula on the growth of a reutericyclin-producing *Enterococcus faecalis*. Log colony-forming units (CFU)/ml are shown for each treatment. Starting inoculum was 10^5^/ml. Samples were cultured with shaking at 200 RPM for 24 hours. Data represent mean ± SD. Student’s *t* test was used to determine significant differences in means; NS = Not Significant.

We next tested the effect of whole human and bovine milk and infant formula on the growth of an *Enterococcus faecalis* strain, essentially a negative control for inhibitory activity, (Fig. 3) obtained from a vaginal swab from a woman with pure culture^22^ of this organism. The strain produces reutericyclin, which may be an analogue of GML^22^. The growth of this organism was not inhibited by human or bovine milk, or infant formula. The stationary phase of growth for this organism in excellent growth medium (Todd Hewitt) was 5.0 × 10^8^/ml. Based on the data obtained, the failure to inhibit the growth of *Enterococcus faecalis* by the milk sample provides evidence that GML in human milk is contributing to the antimicrobial activity against some but not all bacteria, and additionally shows that human milk can support the growth of some bacteria. This demonstrates that human milk is not nutritionally-deficient, but instead is actively antimicrobial.

### Effect of removal of GML and other lipids by ethanol extraction

**Figure 4:**
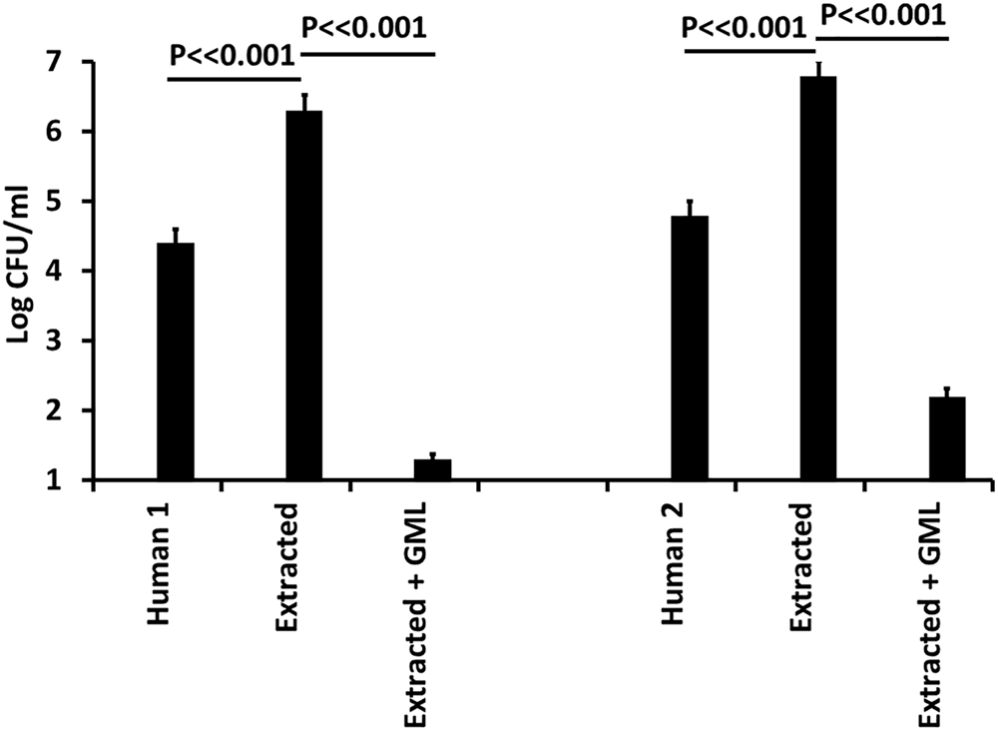
Effect of GML removal from two whole human milk samples (human 1, human 2) on growth of *Staphylococcus aureus* MN8, and effect of re-addition of 3000 µg/ml of GML. Extracted + GML refers to the resolubilized precipitates +3000 µg/ml GML. Log colony-forming units (CFUs)/ml are shown for each treatment. Starting bacterial inoculum was 4.9 × 10^4^/ml. Data represent means ± SD. Student’s *t* test was used to determine significant differences in means. P ≪ 0.001 indicates mean differences much greater than p < 0.001.

We treated two human milk samples with 80% ethanol to precipitate molecules >10,000 molecular weight and leave GML and other soluble lipophilic molecules in the ethanol fraction. The precipitates were resolubilized with distilled water. We then tested the GML-free milk samples, containing 0.03 M glucose, an amount below the standard amount present in human milk in the form of lactose^36^. When GML and other lipophilic molecules were removed from the whole human milk samples, the antimicrobial activity against *S. aureus* was also removed (Fig. 4). We then added back 500 or 3000 µg/ml, amounts of highly-pure, food-grade GML that has been used by us in multiple studies on humans^17,25^; the 3000 µg/ml amount was designed in this experiment to overcome GML reduction in activity by human albumin^29^. Antimicrobial activity was restored by addition of 3000 µg/ml (Fig. 4) but not by addition of 500 µg/ml (data not shown). The fact that 3000 µg/ml GML add-back was more strongly bactericidal after ethanol extraction of human milk, suggests that additional nutrients besides glucose or antimicrobial factors were removed by the extraction process.

### *S. aureus* inhibitory activity of bovine milk after addition of GML

**Figure 5:**
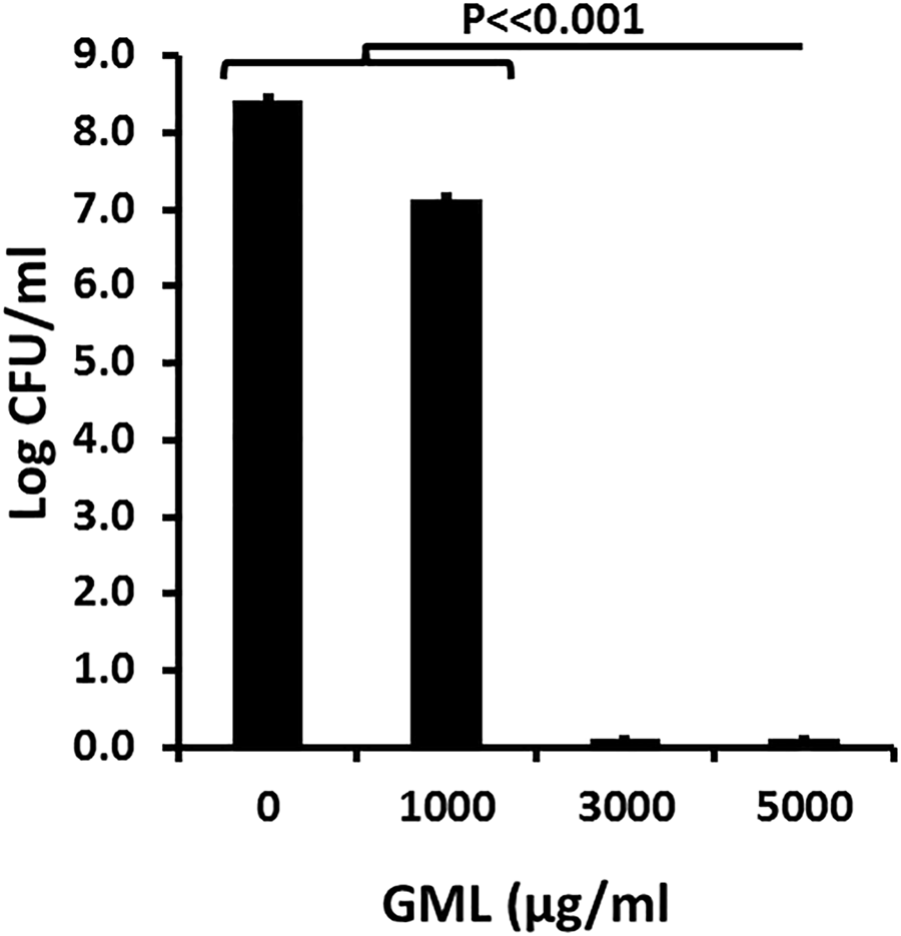
Dose response of GML (0–5000 µg/ml) added to bovine milk samples. Log colony-forming units/ml are shown for all samples. Starting inoculum was approximately 10^5^/ml of bacteria. Samples incubated with shaking (200 RPM) for 24 hour at 37 °C. Data represent means ± SD. Student’s *t* test was used to determine significant differences in means. P ≪ 0.001 indicates mean differences much greater than p < 0.001.

In Fig. 5, we examined the antimicrobial activity of bovine milk after addition of 0–5000 µg/ml of highly-pure GML. Whole bovine milk without GML addition was not antimicrobial. However, successive increases in GML amounts added resulted in antimicrobial activity, with milk containing 3000 and 5000 µg/ml having potent bactericidal activity.

### Anti-inflammatory properties of human milk but not bovine milk

**Figure 6:**
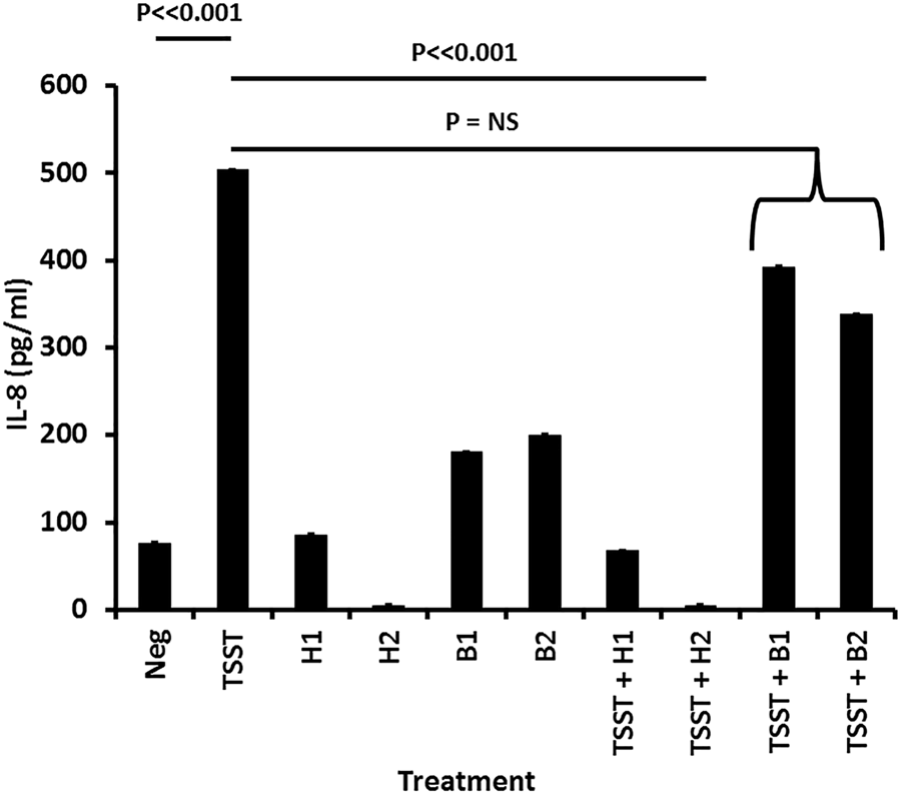
Anti-inflammatory activity of two whole human milk samples versus two whole bovine milk samples during stimulation of human squamous epithelial cells with the superantigen toxic shock syndrome toxin-1 (TSST). Data represent means ± SD of secreted chemokine IL-8, as the representative cytokine, after 6 hours incubation. Assays performed in triplicate in 96 well microtiter plates with confluent human epithelial cells + 200 µl keratinocyte serum-free medium with or without 20 µl milk samples. Student’s *t* test was used to determine significant differences in means. P ≪ 0.001 indicates mean differences much greater than p < 0.001; NS = Not Significant.

In addition to its antimicrobial activity, GML prevents harmful inflammation initiated by pathogens on mucosal surfaces^16,18–21,25^. Also, human milk has been used topically to help in the treatment of atopic dermatitis in children^37^. We thus hypothesized that whole human milk samples, but not whole bovine milk samples, have anti-inflammatory activities against epithelial cells. This was tested on confluent layers of human squamous epithelial cells (HSECs) used only as the model cell system (Fig. 6). In this model test system, as expected the superantigen toxic shock syndrome toxin-1 (TSST-1) caused significant production (in 6 hours) of the chemokine IL-8, as only one of TSST-1 induced cytokines from HSECs^38^. The two representative human milk samples alone added to the HSECs did not induce production of IL-8, but the bovine samples alone caused low-level IL-8 production, but not to the extent of TSST-1. When the two separate human milk samples were added to HSECs in the presence of TSST-1, there was minimal IL-8 produced, indicating both samples were anti-inflammatory. The HSECs alone were not killed by the human or bovine milk samples. Neither bovine sample reduced IL-8 production caused by TSST-1. This experiment was repeated one additional time with similar results. The ethanol-extracted milk samples shown in Fig. 4 lost ability to reduce IL-8 production by TSST-1 (data not shown). We did not fractionate the ethanol-extracted fraction to determine which component(s) were removed that resulted in loss of activity. However, it is likely that GML and other ethanol-soluble molecules contributed to anti-inflammatory activity of human milk samples.

Human milk samples were also tested for ability to reduce IL-8 production from HSECs by the studied bacteria. These bacteria had previously been shown in our laboratory, some published and some unpublished, to stimulate IL-8 production from the HSECs over the 6 hour time period^16,22,30^. For all microbes, human milk reduced the IL-8 production to or near to background.

Collectively, our data suggest that human milk is both inhibitory to pathogen growth and exhibits anti-inflammatory activity with both activities in part dependent on GML. In contrast, bovine milk and commercial formula lacked or had greatly reduced GML and exhibited neither antimicrobial nor anti-inflammatory properties.

## Discussion

It has long been known that breast feeding is highly beneficial to babies through antimicrobial and anti-inflammatory activities. Human milk fed babies select for bifidobacteria, lactobacilli, and enterococci, with limited microbial diversity^6,7^. These organisms are fermenters, producing primarily lactic acid, to some extent acetic acid, and hydrogen peroxide. These organisms plus many components of human milk help keep out potentially pathogenic bacteria. Bovine and commercial formula-fed infants in general have more microbial diversity such that bifidobacteria, lactobacilli, and enterococci numbers are reduced relatively^39,40^.

It is our experience that approximately 1 in 100 women have pure cultures of lactobacilli vaginally (in one case *Enterococcus faecalis*), with no other flora present^22^. These women stably maintain this mono-flora colonization. The lactobacilli and *Enterococcus faecalis* produce antimicrobial compounds, including a broadly antimicrobial and anti-inflammatory compound, reutericyclin, which is a quorum-sensing growth stimulant for these reutericyclin-producing bacteria^23,41^. At the same time, the bacteria have an immunity gene that protects them from reutericyclin^41^.

These observations led us to hypothesize that human breast milk may have an analogue of reutericyclin to cause the predominance of bifidobacteria, lactobacilli, and enterotococci in the gastrointestinal tract. In multiple prior studies we have shown that GML acts as an analogue of reutericyclin, with the same broad spectrum of antimicrobial activity, functioning as a quorum-sensing growth stimulant for lactobacilli, and having anti-inflammatory activity^16–21,25^. The mechanism of bacterial killing by GML, like reutericyclin, appears to be dissipation of the potential difference across bacteria plasma membranes^21^.

In the current study, we first observed that whole pasteurized human milk contains large amounts of GML, compared to 20-fold lesser amounts in whole pasteurized bovine milk. Milk in general contains albumin that potentially interferes with GML activity^29^. However, the amount of GML in human milk samples exceeds the interference by albumin, and additionally human milk contains many other antimicrobial compounds that may synergize with GML. This must be the case since *E. coli*, for example, is not susceptible to GML alone^35^. Thus, we suggest that a significant part of the antimicrobial activity we saw in whole human milk against tested bacteria results from GML alone and in concert with other antimicrobials. Enhancement of GML activity by other antimicrobials has been observed previously^42,43^. Other investigators have also shown that free fatty acids have antimicrobial activity^44–46^. For example, Kabara and colleagues showed that among saturated free fatty acids, lauric acid, the fatty acid contained in GML, has the greatest antimicrobial activity, with the same bacterial inhibitory spectrum as GML^45^. We have recently duplicated these findings, but our studies showed that GML is nearly 400-fold more active against *S. aureus* than lauric acid^21^. Indeed, Kabara and colleagues, who did much of the pioneering related to GML, later focused many of their studies on GML because of its increased activity^46^. Despite these findings, it is important to consider that human milk contains multiple other antimicrobial compounds that may synergize or act additively with GML.

Although we do not know why human milk contains so much GML, compared to bovine milk, this observation is supported by based differences in gastrointestinal tracts of humans and cows. Cows have four stomachs that must adapt to processing grasses and related plants through cellulases. This means cows must also have a quite different gut microbiome than humans, one that may depend on not having high concentrations of GML.

Most microbial pathogens stimulate harmful inflammation at mucosal surfaces^17,19–21,25^. We have previously provided strong evidence that the superantigen, TSST-1, as studied in the current manuscript, and simian immunodeficiency virus (SIV) cause harmful inflammation at such mucosal surfaces^17,19–21,25^. The net effect is to open the mucosal barrier to TSST-1 to result in menstrual TSS, and SIV to result in infection, and to attract innate and adaptive immune cells that ultimately are the direct targets for disease causation. In this manuscript, we show that whole human milk samples are anti-inflammatory as tested with IL-8 production, whereas ethanol-extracted human milk and whole bovine milk samples are not. While the significance of this is incompletely known, the data are consistent with slowing but not stopping development of harmful pathogen responses so long as babies are breast-fed^39,40^. It is recognized that intestinal microbiome organisms are essential for immune system development. However, over-reactivity may lead to harmful effects, such as asthma and atopic dermatitis.

Collectively, our study suggests that there are great benefits to human milk compared to bovine milk and formula. Positive effects of human milk appear to be due in part to the presence of GML combined with other known and unknown factors^39,40^. Future studies are needed to determine whether or not supplementation of bovine milk and commercial infant formula with GML will be beneficial.

## Methods

All experiments with use of whole human milk and use of human epithelial cells were performed in accordance with accepted regulatory requirements. The human epithelial cells have been used by our laboratory in multiple publications since 2005^38^. University of Iowa Institutional Review Board approval and consent (approval number 199910006) were obtained prior to obtaining and using these cells. The human milk samples were purchased from Mother’s Milk Bank of Iowa, Coralville, IA.; IRB approval was not required for use of these samples. The milk samples were not identified as to donor.

### Bacteria and TSST-1

*Staphylococcus aureus* MN8 is a commonly used strain from a patient with menstrual TSS, but this clonal group of *S. aureus* is also associated with enterocolitis^32,33,38^. *B. subtilis*^34^, *C. perfringens*^34^, and *E. coli*^21^ are Schlievert laboratory strains of stock cultures. *Enterococcus faecalis* was obtained from a vaginal culture from a woman with pure culture of this organism; the strain produces reutericyclin^22^. These organisms are all maintained as −80 °C frozen cultures. We chose these strains for study to include bacteria that are considered typically highly susceptible to GML (Gram-positives) and bacteria that are typically resistant to GML (Gram-negative *E. coli*). However, we have shown that the reutericyclin-producing *Enterococcus faecalis* strain (Gram-positive) is resistant to GML^22^. Prior to addition of microbes to milk samples, the organisms were cultured overnight in Todd Hewitt (Difco, Detroit, MI) broths. The aerobic organisms were cultured at 37 °C with aeration (shaking at 200 RPM); *C. perfringens* was cultured stationary in an anaerobic chamber. Subsequently, each organism was diluted to the desired starting inoculum for addition to milk samples.

The superantigen TSST-1 was purified after culture of *S. aureus* RN6390 containing the *tstH* gene on plasmid pCE107^47^. TSST-1 was purified by ethanol precipitation from cultures followed by isoelectric focusing^48,49^. Toxin thus purified is homogeneous when tested by SDS-PAGE^48,49^.

### Whole human and bovine milk and commercial infant formula samples

In all studies, for consistency, milk sample were pasteurized either by us or prior to their purchase from the commercial providers. Non-purchased samples were pasteurized by incubation at 65 °C for 30 minutes. Whole human milk samples were purchased from the Mother’s Milk Bank of Iowa, Coralville, IA. Whole bovine milk samples were obtained from cows at the dairy center at Mississippi State University by Dr. Keun Seok Seo. Commercially-available, previously pasteurized, bovine milk was purchased from HyVee Grocery Store, Coralville, IA. Infant formula was Similac Advance, also purchased from HyVee; this infant formula is a product of Abbott Laboratories, Columbus, OH. The formula was diluted two-fold with sterile distilled water as directed by the manufacturer. Milk fat was not removed from any samples, but instead was thoroughly mixed in the milk samples.

### Glycerol monolaurate (GML)

Highly-pure, human, food grade GML was purchased from Colonial, South Pittsburgh, TN. The pellets were solubilized in absolute ethanol as a stock culture at 100 mg/ml for addition to milk samples. GML in milk samples was measured by mass spectrometry on a fee-for-service basis at the University of Iowa Metabolomics Center, compared to known standards. GML concentrations in milk samples were considered validated after addition of 3000 μg/ml of purified GML to bovine milk demonstrated of antimicrobial activity against *S. aureus*, and thus overcoming albumin’s ability to interfere with GML activity^29^. GML and other lipophilic compounds were removed from human milk samples by treatment with ethanol (80% final concentration) to precipitate milk molecules >10,000 molecular weight. The precipitates were dried and then resolubilized in distilled water to the original volume.

### Human squamous epithelial cells

We used human squamous epithelial cells (HSECs) as a model system^38^ to test if human and bovine milk samples exhibit anti-inflammatory activity. These gently immortalized vaginal epithelial cells were cultured in triplicate to confluence in 96 well, flat-bottom microtiter plates in keratinocyte serum-free medium. Once confluent, the cells were treated for 6 hours in the absence and presence of 100 µg of the superantigen toxic shock syndrome toxin-1 (TSST-1) and with and without 20 µl of milk samples. The supernates were then tested for the pro-inflammatory chemokine IL-8 by Quantikine kit (R&D systems Minneapolis, MN). The experiment was repeated one additional time. Previously, we have shown that TSST-1 stimulates production of many pro-inflammatory cytokines by HSECs and keratinocytes, including MIP-3α and IL-8, whose genes may be up-regulated 500 and 80-fold, respectively^38^. Our studies were not designed to assess the effects of TSST-1 with and without milk samples on other innate and adaptive immune cells. However, our prior studies showed that a major effect of TSST-1 and SIV was on epithelial cells to produce harmful inflammation, these cells being present in many times the numbers of other immune cells^19,20,22^. Human milk samples alone were tested for resistance to killing by human milk through performing the 6 hour incubations with milk alone and then performing trypan blue dye exclusion. Finally, we tested all stationary phase (grown in Todd Hewitt) bacteria for stimulation of the epithelial cells and inhibition of IL-8 production by human milk in the same types of studies with use of 20 μl of washed bacteria (4000 × g, 10 minutes; resuspended in PBS)^16,22^.

## Statistics

Means, standard deviations, and Student’s *t* test analyses were performed with Microsoft Excel 2010.
